# The genome sequence of the Small China-mark, *Cataclysta lemnata* (Linnaeus, 1758) (Lepidoptera: Crambidae)

**DOI:** 10.12688/wellcomeopenres.25182.1

**Published:** 2025-11-21

**Authors:** David C. Lees, Natural History Museum Genome Acquisition Lab, Darwin Tree of Life Barcoding Collective, Wellcome Sanger Institute Tree of Life Management, Samples and Laboratory team, Wellcome Sanger Institute Scientific Operations: Sequencing Operations, Wellcome Sanger Institute Tree of Life Core Informatics team, Tree of Life Core Informatics collective, Darwin Tree of Life Consortium

**Affiliations:** 1 Natural History Museum, London, England, UK

**Keywords:** Cataclysta lemnata; Small China-mark; genome sequence; chromosomal; Lepidoptera

## Abstract

We present a genome assembly from an individual male *Cataclysta lemnata* (Small China-mark; Arthropoda; Insecta; Lepidoptera; Crambidae). The genome sequence has a total length of 432.44 megabases. Most of the assembly (99.51%) is scaffolded into 28 chromosomal pseudomolecules, including the Z sex chromosome. The mitochondrial genome has also been assembled, with a length of 15.36 kilobases. This assembly was generated as part of the Darwin Tree of Life project, which produces reference genomes for eukaryotic species found in Britain and Ireland.

## Species taxonomy

Eukaryota; Opisthokonta; Metazoa; Eumetazoa; Bilateria; Protostomia; Ecdysozoa; Panarthropoda; Arthropoda; Mandibulata; Pancrustacea; Hexapoda; Insecta; Dicondylia; Pterygota; Neoptera; Endopterygota; Amphiesmenoptera; Lepidoptera; Glossata; Neolepidoptera; Heteroneura; Ditrysia; Obtectomera; Pyraloidea; Crambidae; Nymphulinae; *Cataclysta*; *Cataclysta lemnata* (Linnaeus, 1758) (NCBI:txid1594245)

## Background

*Cataclysta lemnata* (Linnaeus, 1758), known in Britain as the Small China-mark, is a moth in the family Crambidae, belonging to the largely aquatic subfamily Acentropinae. It is sexually dimorphic. The male has a forewing length of 8–11 mm ( Sterling *et al.*, 2023), and the wingspan is about 13–18 mm in males and 18–24 mm in females ( Farahpour-Haghani *et al.*, 2017). The male forewing is glossy white with a post-median black discal spot, and the hindwing has a dark discal crescent bordered by irregular narrow brown lines and a black margin containing about 6–7 white glittery eyespots; the female is yellowish-brown, with the same ocelli ( Sterling *et al.*, 2023). These eyespots are often displayed at rest when the moth adopts a shallowly tented triangular posture and has a general resemblance to the eyes of salticid spiders ( Hill, 2022: 15). The moth flies from May to late September, with a peak in Britain in July and August ( Norfolk Moths, 2025). The male flies crepuscularly over the water surface, while females are nocturnal ( Catalogue of the Lepidoptera of Belgium, 2024). Both sexes, especially females, are attracted to light.

The Small China-mark occurs in freshwater habitats including ponds and lakes ( Sterling *et al.*, 2023). The oval larval case made of leaf fragments contains bubbles which allow the larva to breathe and the white oval cocoon is attached to leaves just below the water surface ( Catalogue of the Lepidoptera of Belgium, 2024; Marković *et al.*, 2024).

There are currently 3 568 records of *C. lemnata* on the NBN Atlas Partnership (2025) and it is widely distributed in England, and Ireland ( Moths Ireland, 2025). In Scotland it occurs mainly in the southwest ( Scottish Micro Moth Distribution Maps, 2025); it also occurs along the eastern seaboard of Ireland ( Moths Ireland, 2025). There are 24 039 records ( GBIF Secretariat, 2025), distributed from Ireland through Europe as far east as Lake Baikal and from southern Scandinavia to the shores of the Mediterranean, Spain, Crete and the Caspian Sea. It is one of the most abundant Palaearctic aquatic moths ( Marković *et al.*, 2024).

*C. lemnata* larvae feed particularly on Duckweed ( *Lemna* L., Araceae) ( Marković *et al.*, 2024) and have been recently found to feed on Water ferns ( *Azolla* Lam., Azollaceae) in Iran ( Farahpour-Haghani *et al.*, 2017). They can eat other aquatic macrophytes such as *Stratiotes aloides* L., *Typha latifolia* L. and *Glyceria maxima* (Hartm.) Holmb. ( van der Velde, 1988). The early stages, first studied in detail by Chapman (1905) are described and illustrated in Farahpour-Haghani *et al.* (2017).

On BOLD (accessed 14/10/2025) the DNA barcode from the mitochondrial assembly (OZ008366.1) represents the Barcode Index Number (BIN) BOLD:AAC2080, with limited haplotype variability (maximum 0.64%, *n* = 53) and identical haplotypes occurring in Europe. This cluster is remote (a *p*-distance of over 9%) from other acentropine crambids on BOLD. Hawking (2014) considered it different from seven other species of *Cataclysta*, which may not merit belonging in the same genus.

TWe present a chromosomally complete genome sequence for *C. lemnata*. The genome will be useful for investigating the phylogenetic relationships of *C. lemnata* and adaptations to aquatic life ( Pabis, 2018), particularly in its early stages. Their larvae can eat holes in PVC foil, providing another example of Lepidoptera that can be used in studies of plastic waster degradation ( van der Velde, 1991). It may well also be useful in further studies of the properties of underwater silk ( Heckenhauer *et al.*, 2024).

**Figure 1.**
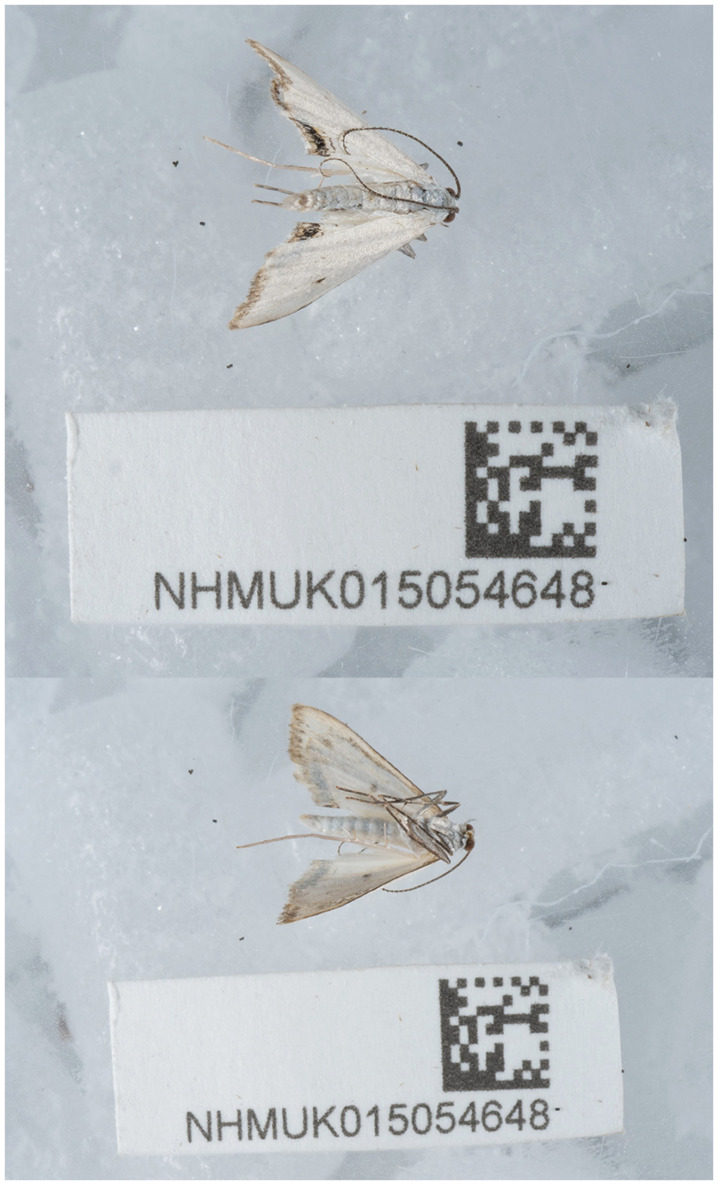
Photographs of the *Cataclysta lemnata* (ilCatLemn2) specimen used for genome sequencing.

The assembly was produced using the Tree of Life pipeline from a specimen collected in Winterton Dunes, England, UK ( Figure 1), as part of the Darwin Tree of Life project.

## Methods

### Sample acquisition and DNA barcoding

The specimen used for genome sequencing was an adult male *Cataclysta lemnata* (specimen ID NHMUK015054648, ToLID ilCatLemn2; Figure 1), collected from Winterton Dunes, England, United Kingdom (latitude 52.73, longitude 1.69) on 2022-06-29. The specimen was collected and identified by David Lees (Natural History Museum).

The initial identification was verified by an additional DNA barcoding process according to the framework developed by Twyford *et al.* (2024). A small sample was dissected from the specimen and stored in ethanol, while the remaining parts were shipped on dry ice to the Wellcome Sanger Institute (WSI) (see the protocol). The tissue was lysed, the COI marker region was amplified by PCR, and amplicons were sequenced and compared to the BOLD database, confirming the species identification ( Crowley *et al.*, 2023). Following whole genome sequence generation, the relevant DNA barcode region was also used alongside the initial barcoding data for sample tracking at the WSI ( Twyford *et al.*, 2024). The standard operating procedures for Darwin Tree of Life barcoding are available on protocols.io.

Sample metadata were collected in line with the Darwin Tree of Life project standards described by Lawniczak *et al*. (2022).

### Nucleic acid extraction

Protocols for high molecular weight (HMW) DNA extraction developed at the Wellcome Sanger Institute (WSI) Tree of Life Core Laboratory are available on protocols.io ( Howard *et al.*, 2025). The ilCatLemn2 sample was weighed and triaged to determine the appropriate extraction protocol. Tissue from the head and thorax was homogenised by powermashing using a PowerMasher II tissue disruptor. HMW DNA was extracted in the WSI Scientific Operations core using the Automated MagAttract v2 protocol. We used centrifuge-mediated fragmentation to produce DNA fragments in the 8–10 kb range, following the Covaris g-TUBE protocol for ultra-low input (ULI). Sheared DNA was purified by automated SPRI (solid-phase reversible immobilisation). The concentration of the sheared and purified DNA was assessed using a Nanodrop spectrophotometer and Qubit Fluorometer using the Qubit dsDNA High Sensitivity Assay kit. Fragment size distribution was evaluated by running the sample on the FemtoPulse system.

### PacBio HiFi library preparation and sequencing

Library preparation and sequencing were performed at the WSI Scientific Operations core. Prior to library preparation, the DNA was fragmented to ~10 kb. Ultra-low-input (ULI) libraries were prepared using the PacBio SMRTbell® Express Template Prep Kit 2.0 and gDNA Sample Amplification Kit. Samples were normalised to 20 ng DNA. Single-strand overhang removal, DNA damage repair, and end-repair/A-tailing were performed according to the manufacturer’s instructions, followed by adapter ligation. A 0.85× pre-PCR clean-up was carried out with Promega ProNex beads.

The DNA was evenly divided into two aliquots for dual PCR (reactions A and B), both following the manufacturer’s protocol. A 0.85× post-PCR clean-up was performed with ProNex beads. DNA concentration was measured using a Qubit Fluorometer v4.0 (Thermo Fisher Scientific) with the Qubit HS Assay Kit, and fragment size was assessed on an Agilent Femto Pulse Automated Pulsed Field CE Instrument (Agilent Technologies) using the gDNA 55 kb BAC analysis kit. PCR reactions A and B were then pooled, ensuring a total mass of ≥500 ng in 47.4 μl.

The pooled sample underwent another round of DNA damage repair, end-repair/A-tailing, and hairpin adapter ligation. A 1× clean-up was performed with ProNex beads, followed by DNA quantification using the Qubit and fragment size analysis using the Agilent Femto Pulse. Size selection was performed on the Sage Sciences PippinHT system, with target fragment size determined by Femto Pulse analysis (typically 4–9 kb). Size-selected libraries were cleaned with 1.0× ProNex beads and normalised to 2 nM before sequencing.

The sample was sequenced using the Sequel IIe system (Pacific Biosciences, California, USA). The concentration of the library loaded onto the Sequel IIe was in the range 40–135 pM. The SMRT link software, a PacBio web-based end-to-end workflow manager, was used to set-up and monitor the run, and to perform primary and secondary analysis of the data upon completion.

### Hi-C

***Sample preparation and crosslinking***

The Hi-C sample was prepared from 20–50 mg of frozen head and thorax tissue of the ilCatLemn2 sample using the Arima-HiC v2 kit (Arima Genomics). Following the manufacturer’s instructions, tissue was fixed and DNA crosslinked using TC buffer to a final formaldehyde concentration of 2%. The tissue was homogenised using the Diagnocine Power Masher-II. Crosslinked DNA was digested with a restriction enzyme master mix, biotinylated, and ligated. Clean-up was performed with SPRISelect beads before library preparation. DNA concentration was measured with the Qubit Fluorometer (Thermo Fisher Scientific) and Qubit HS Assay Kit. The biotinylation percentage was estimated using the Arima-HiC v2 QC beads.

***Hi-C library preparation and sequencing***

Biotinylated DNA constructs were fragmented using a Covaris E220 sonicator and size selected to 400–600 bp using SPRISelect beads. DNA was enriched with Arima-HiC v2 kit Enrichment beads. End repair, A-tailing, and adapter ligation were carried out with the NEBNext Ultra II DNA Library Prep Kit (New England Biolabs), following a modified protocol where library preparation occurs while DNA remains bound to the Enrichment beads. Library amplification was performed using KAPA HiFi HotStart mix and a custom Unique Dual Index (UDI) barcode set (Integrated DNA Technologies). Depending on sample concentration and biotinylation percentage determined at the crosslinking stage, libraries were amplified with 10–16 PCR cycles. Post-PCR clean-up was performed with SPRISelect beads. Libraries were quantified using the AccuClear Ultra High Sensitivity dsDNA Standards Assay Kit (Biotium) and a FLUOstar Omega plate reader (BMG Labtech).

Prior to sequencing, libraries were normalised to 10 ng/μL. Normalised libraries were quantified again and equimolar and/or weighted 2.8 nM pools were created. Pool concentrations were checked using the Agilent 4200 TapeStation (Agilent) with High Sensitivity D500 reagents before sequencing. Sequencing was performed using paired-end 150 bp reads on the Illumina NovaSeq 6000.

### Genome assembly

Prior to assembly of the PacBio HiFi reads, a database of *k*-mer counts ( *k* = 31) was generated from the filtered reads using FastK. GenomeScope2 ( Ranallo-Benavidez *et al.*, 2020) was used to analyse the *k*-mer frequency distributions, providing estimates of genome size, heterozygosity, and repeat content.

The HiFi reads were assembled using Hifiasm ( Cheng *et al.*, 2021) with the --primary option. Haplotypic duplications were identified and removed using purge_dups ( Guan *et al.*, 2020). The Hi-C reads ( Rao *et al.*, 2014) were mapped to the primary contigs using bwa-mem2 ( Vasimuddin *et al.*, 2019), and the contigs were scaffolded in YaHS ( Zhou *et al.*, 2023) with the --break option for handling potential misassemblies. The scaffolded assemblies were evaluated using Gfastats ( Formenti *et al.*, 2022), BUSCO ( Manni *et al.*, 2021) and MERQURY.FK ( Rhie *et al.*, 2020).

The mitochondrial genome was assembled using MitoHiFi ( Uliano-Silva *et al.*, 2023), which runs MitoFinder ( Allio *et al.*, 2020) and uses these annotations to select the final mitochondrial contig and to ensure the general quality of the sequence.

### Assembly curation

The assembly was decontaminated using the Assembly Screen for Cobionts and Contaminants ( ASCC) pipeline. TreeVal was used to generate the flat files and maps for use in curation. Manual curation was conducted primarily in PretextView and HiGlass ( Kerpedjiev *et al.*, 2018). Scaffolds were visually inspected and corrected as described by Howe *et al*. (2021). Manual corrections included 46 breaks, 88 joins, and removal of 24 haplotypic duplications. The curation process is documented at https://gitlab.com/wtsi-grit/rapid-curation. PretextSnapshot was used to generate a Hi-C contact map of the final assembly.

### Assembly quality assessment

The Merqury.FK tool ( Rhie *et al.*, 2020) was run in a Singularity container ( Kurtzer *et al.*, 2017) to evaluate *k*-mer completeness and assembly quality for the primary and alternate haplotypes using the *k*-mer databases ( *k* = 31) computed prior to genome assembly. The analysis outputs included assembly QV scores and completeness statistics.

The genome was analysed using the BlobToolKit pipeline, a Nextflow implementation of the earlier Snakemake version ( Challis *et al.*, 2020). The pipeline aligns PacBio reads using minimap2 ( Li, 2018) and SAMtools ( Danecek *et al.*, 2021) to generate coverage tracks. It runs BUSCO ( Manni *et al.*, 2021) using lineages identified from the NCBI Taxonomy ( Schoch *et al.*, 2020). For the three domain-level lineages, BUSCO genes are aligned to the UniProt Reference Proteomes database ( Bateman *et al.*, 2023) using DIAMOND blastp ( Buchfink *et al.*, 2021). The genome is divided into chunks based on the density of BUSCO genes from the closest taxonomic lineage, and each chunk is aligned to the UniProt Reference Proteomes database with DIAMOND blastx. Sequences without hits are chunked using seqtk and aligned to the NT database with blastn ( Altschul *et al.*, 1990). The BlobToolKit suite consolidates all outputs into a blobdir for visualisation. The BlobToolKit pipeline was developed using nf-core tooling ( Ewels *et al.*, 2020) and MultiQC ( Ewels *et al.*, 2016), with containerisation through Docker ( Merkel, 2014) and Singularity ( Kurtzer *et al.*, 2017).

## Genome sequence report

### Sequence data

**Figure 2.**
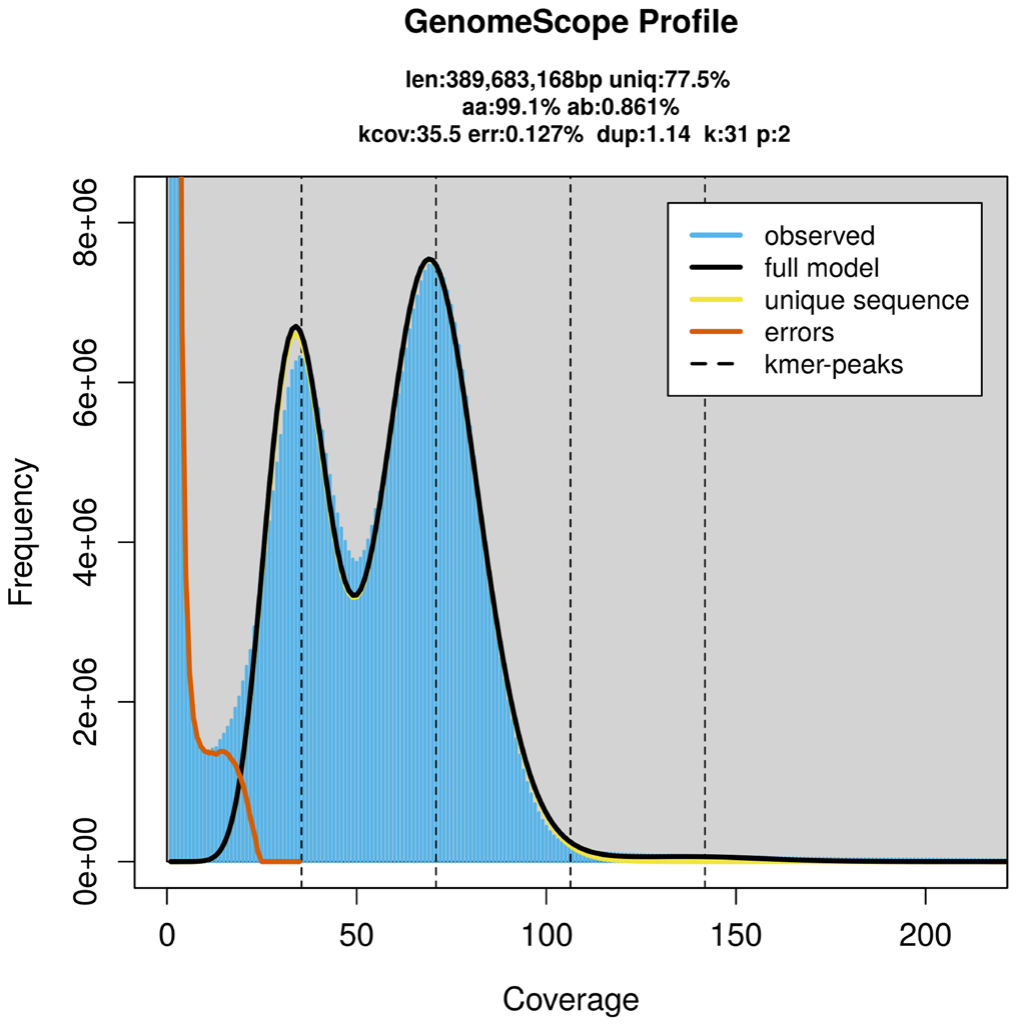
Frequency distribution of *k*-mers generated using GenomeScope2. The plot shows observed and modelled *k*-mer spectra, providing estimates of genome size, heterozygosity, and repeat content based on unassembled sequencing reads.

PacBio sequencing of the *Cataclysta lemnata* specimen generated 29.41 Gb (gigabases) from 2.70 million reads, which were used to assemble the genome. GenomeScope2.0 analysis estimated the haploid genome size at 389.68 Mb, with a heterozygosity of 0.86% and repeat content of 22.63% ( Figure 2). These estimates guided expectations for the assembly. Based on the estimated genome size, the sequencing data provided approximately 71× coverage. Hi-C sequencing produced 134.27 Gb from 889.23 million reads, which were used to scaffold the assembly. Table 1 summarises the specimen and sequencing details.

**Table 1.** Specimen and sequencing data for BioProject PRJEB72119.

Platform	PacBio HiFi	Hi-C
**ToLID**	ilCatLemn2	ilCatLemn2
**Specimen ID**	NHMUK015054648	NHMUK015054648
**BioSample (source ** **individual)**	SAMEA112963103	SAMEA112963103
**BioSample (tissue)**	SAMEA112963199	SAMEA112963199
**Tissue**	head and thorax	head and thorax
**Instrument**	Sequel IIe	Illumina NovaSeq 6000
**Run accessions**	ERR12535459	ERR12543000
**Read count total**	2.70 million	889.23 million
**Base count total**	29.41 Gb	134.27 Gb

### Assembly statistics

The primary haplotype was assembled, and contigs corresponding to an alternate haplotype were also deposited in INSDC databases. The final assembly has a total length of 432.44 Mb in 137 scaffolds, with 874 gaps, and a scaffold N50 of 15.95 Mb ( Table 2).

**Table 2.** Genome assembly statistics.

**Assembly name**	ilCatLemn2.1
**Assembly accession**	GCA_963931975.1
**Alternate haplotype accession**	GCA_963931965.1
**Assembly level**	chromosome
**Span (Mb)**	432.44
**Number of chromosomes**	28
**Number of contigs**	1 011
**Contig N50**	0.99 Mb
**Number of scaffolds**	137
**Scaffold N50**	15.95 Mb
**Sex chromosomes**	Z
**Organelles**	Mitochondrion: 15.36 kb

**Figure 3.**
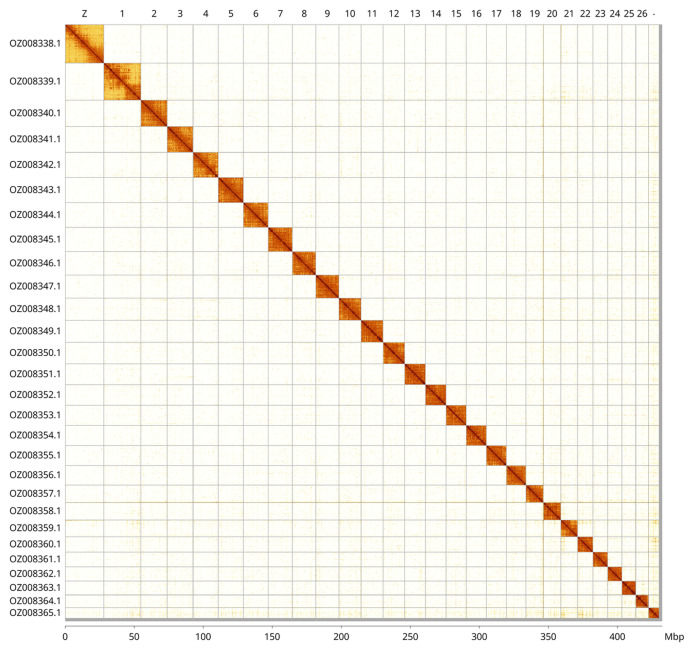
Hi-C contact map of the *Cataclysta lemnata* genome assembly. Assembled chromosomes are shown in order of size and labelled along the axes, with a megabase scale shown below. The plot was generated using PretextSnapshot.

Most of the assembly sequence (99.51%) was assigned to 28 chromosomal-level scaffolds, representing 27 autosomes and the Z sex chromosome. These chromosome-level scaffolds, confirmed by Hi-C data, are named according to size ( Figure 3; Table 3). Z chromosome identified based on alignment with the genome of *Nymphula nitidulata* (GCA_947347705.1).

**Table 3.** Chromosomal pseudomolecules in the primary genome assembly of *Cataclysta lemnata* ilCatLemn2.

INSDC accession	Molecule	Length (Mb)	GC%
OZ008339.1	1	26.92	37.50
OZ008340.1	2	19.10	37.50
OZ008341.1	3	18.70	37.50
OZ008342.1	4	18.24	38
OZ008343.1	5	18.14	37.50
OZ008344.1	6	18.04	37.50
OZ008345.1	7	17.54	37.50
OZ008346.1	8	16.96	37.50
OZ008347.1	9	16.72	37.50
OZ008348.1	10	16.04	38
OZ008349.1	11	15.95	38
OZ008350.1	12	15.55	38
OZ008351.1	13	15.14	37.50
OZ008352.1	14	14.96	38
OZ008353.1	15	14.60	38
OZ008354.1	16	14.56	38
OZ008355.1	17	14.46	38.50
OZ008356.1	18	14.14	38
OZ008357.1	19	12.71	39.50
OZ008358.1	20	12.55	38
OZ008359.1	21	12.18	39
OZ008360.1	22	11.11	39
OZ008361.1	23	10.66	39
OZ008362.1	24	10.34	39
OZ008363.1	25	10.09	39.50
OZ008364.1	26	9.20	39.50
OZ008365.1	27	7.74	40
OZ008338.1	Z	27.99	37

The mitochondrial genome was also assembled. This sequence is included as a contig in the multifasta file of the genome submission and as a standalone record.

**Figure 4.**
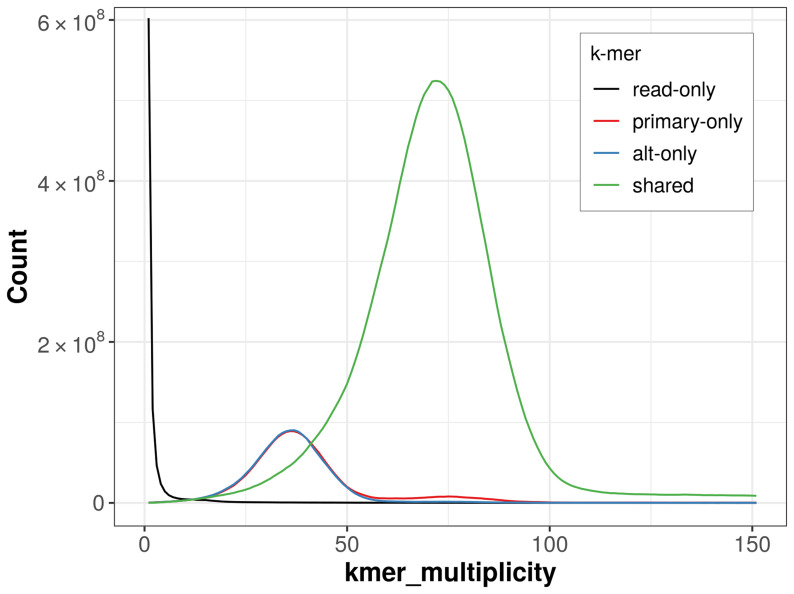
Evaluation of *k*-mer completeness using MerquryFK. This plot illustrates the recovery of *k*-mers from the original read data in the final assemblies. The horizontal axis represents *k*-mer multiplicity, and the vertical axis shows the number of *k*-mers. The black curve represents *k*-mers that appear in the reads but are not assembled. The green curve corresponds to *k*-mers shared by both haplotypes, and the red and blue curves show *k*-mers found only in one of the haplotypes.

The combined primary and alternate assemblies achieve an estimated QV of 59.9. The *k*-mer completeness is 85.17% for the primary assembly, 84.70% for the alternate haplotype, and 99.36% for the combined assemblies ( Figure 4).

**Figure 5.**
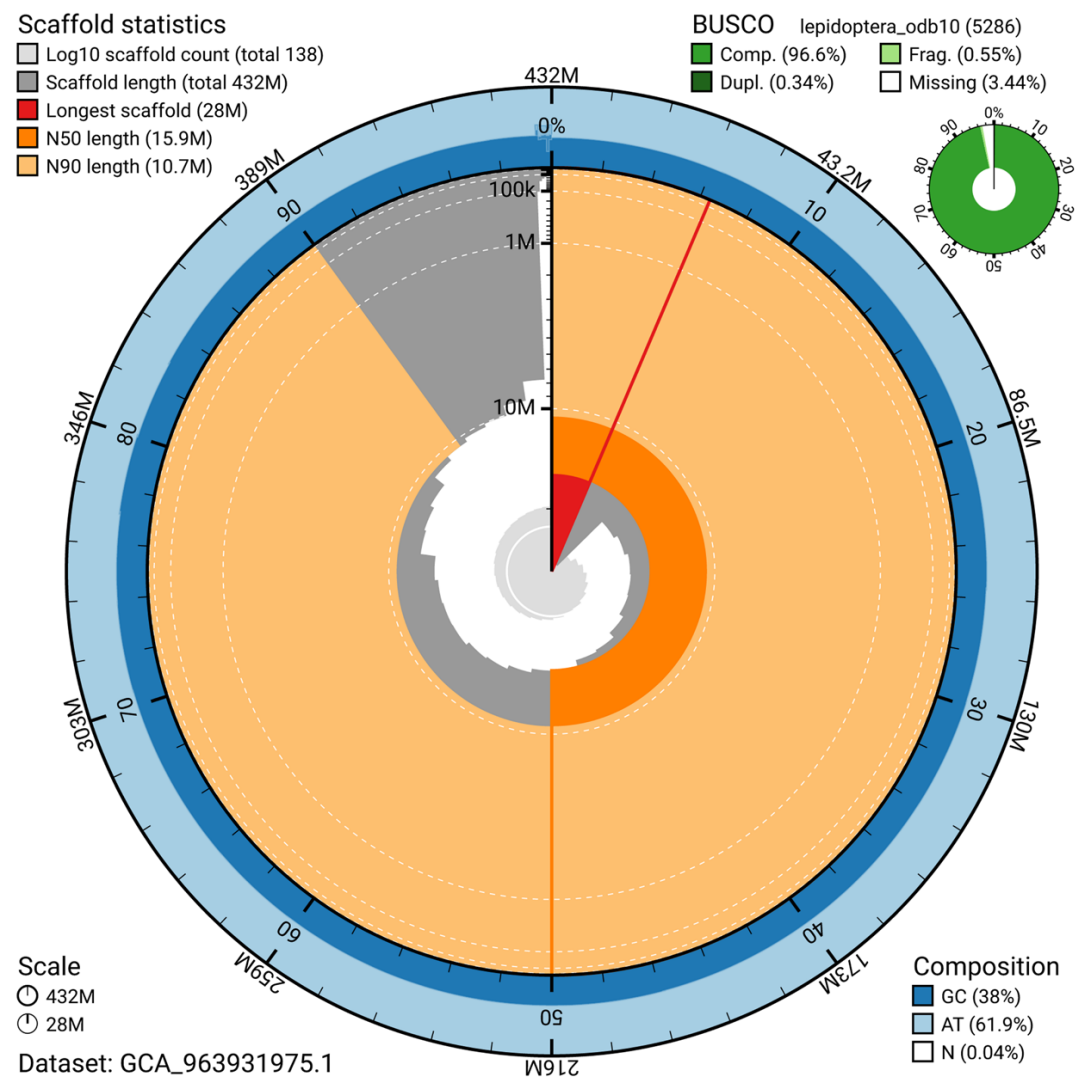
Assembly metrics for ilCatLemn2.1. The BlobToolKit snail plot provides an overview of assembly metrics and BUSCO gene completeness. The circumference represents the length of the whole genome sequence, and the main plot is divided into 1 000 bins around the circumference. The outermost blue tracks display the distribution of GC, AT, and N percentages across the bins. Scaffolds are arranged clockwise from longest to shortest and are depicted in dark grey. The longest scaffold is indicated by the red arc, and the deeper orange and pale orange arcs represent the N50 and N90 lengths. A light grey spiral at the centre shows the cumulative scaffold count on a logarithmic scale. A summary of complete, fragmented, duplicated, and missing BUSCO genes in the lepidoptera_odb10 set is presented at the top right. An interactive version of this figure can be accessed on the BlobToolKit viewer.

**Figure 6.**
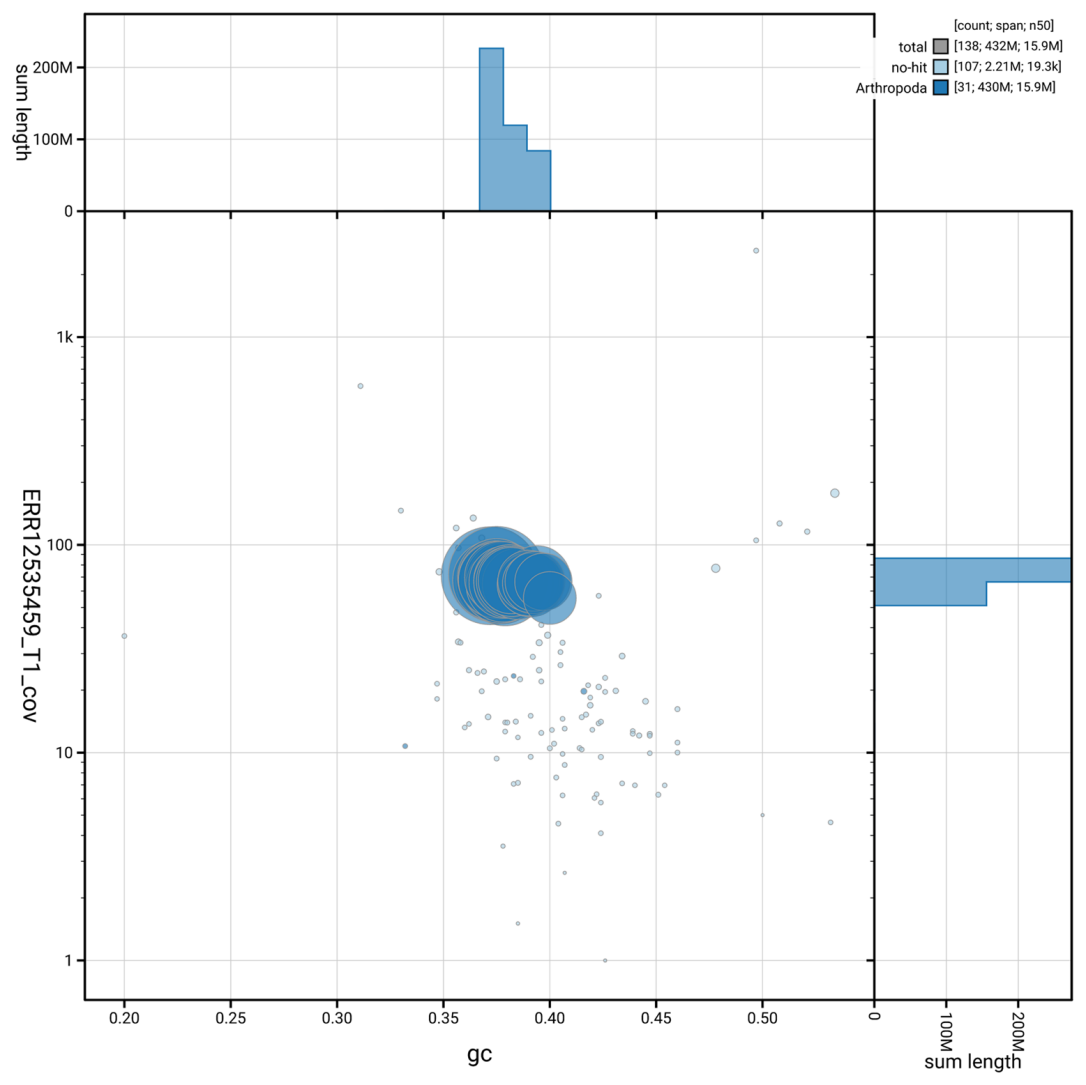
BlobToolKit GC-coverage plot for ilCatLemn2.1. Blob plot showing sequence coverage (vertical axis) and GC content (horizontal axis). The circles represent scaffolds, with the size proportional to scaffold length and the colour representing phylum membership. The histograms along the axes display the total length of sequences distributed across different levels of coverage and GC content. An interactive version of this figure is available on the BlobToolKit viewer.

BUSCO v.5.5.0 analysis using the lepidoptera_odb10 reference set ( *n* = 5 286) identified 96.6% of the expected gene set (single = 96.2%, duplicated = 0.3%). The snail plot in Figure 5 summarises the scaffold length distribution and other assembly statistics for the primary assembly. The blob plot in Figure 6 shows the distribution of scaffolds by GC proportion and coverage.

Table 4 lists the assembly metric benchmarks adapted from Rhie *et al.* (2021) and the Earth BioGenome Project Report on Assembly Standards September 2024. The EBP metric for the primary assembly is **5.C.Q59**.

**Table 4.** Earth Biogenome Project summary metrics for the *Cataclysta lemnata* assembly.

Measure	Value	Benchmark
EBP summary (primary)	5.C.Q59	6.C.Q40
Contig N50 length	0.99 Mb	≥ 1 Mb
Scaffold N50 length	15.95 Mb	= chromosome N50
Consensus quality (QV)	Primary: 59.9; alternate: 60.8; combined: 59.9	≥ 40
*k*-mer completeness	Primary: 85.17%; alternate: 84.70%; combined: 99.36%	≥ 95%
BUSCO	C:96.6% [S:96.2%; D:0.3%]; F:0.5%; M:2.9%; n:5 286	S > 90%; D < 5%
Percentage of assembly assigned to chromosomes	99.51%	≥ 90%

### Genome annotation report

The *Cataclysta lemnata* genome assembly (GCA_963931975.1) was annotated by Ensembl at the European Bioinformatics Institute (EBI). This annotation was done using BRAKER2 and identified 17 004 protein-coding genes. For further information about the annotation, please refer to the Ensembl annotation page.

### Wellcome Sanger Institute – Legal and Governance

The materials that have contributed to this genome note have been supplied by a Darwin Tree of Life Partner. The submission of materials by a Darwin Tree of Life Partner is subject to the **‘Darwin Tree of Life Project Sampling Code of Practice’**, which can be found in full on the Darwin Tree of Life website. By agreeing with and signing up to the Sampling Code of Practice, the Darwin Tree of Life Partner agrees they will meet the legal and ethical requirements and standards set out within this document in respect of all samples acquired for, and supplied to, the Darwin Tree of Life Project. Further, the Wellcome Sanger Institute employs a process whereby due diligence is carried out proportionate to the nature of the materials themselves, and the circumstances under which they have been/are to be collected and provided for use. The purpose of this is to address and mitigate any potential legal and/or ethical implications of receipt and use of the materials as part of the research project, and to ensure that in doing so we align with best practice wherever possible. The overarching areas of consideration are:

- Ethical review of provenance and sourcing of the material
- Legality of collection, transfer and use (national and international)

Each transfer of samples is further undertaken according to a Research Collaboration Agreement or Material Transfer Agreement entered into by the Darwin Tree of Life Partner, Genome Research Limited (operating as the Wellcome Sanger Institute), and in some circumstances, other Darwin Tree of Life collaborators.

## Data Availability

European Nucleotide Archive: Cataclysta lemnata (small china-mark). Accession number PRJEB72119. The genome sequence is released openly for reuse. The *Cataclysta lemnata* genome sequencing initiative is part of the Darwin Tree of Life Project (PRJEB40665), the Sanger Institute Tree of Life Programme (PRJEB43745) and Project Psyche (PRJEB71705). All raw sequence data and the assembly have been deposited in INSDC databases. Raw data and assembly accession identifiers are reported in Table 1 and Table 2. Production code used in genome assembly at the WSI Tree of Life is available at https://github.com/sanger-tol. Table 5 lists software versions used in this study.

## Author information

Contributors are listed at the following links:

- Members of the Natural History Museum Genome Acquisition Lab
- Members of the Darwin Tree of Life Barcoding collective
- Members of the Wellcome Sanger Institute Tree of Life Management, Samples and Laboratory team
- Members of Wellcome Sanger Institute Scientific Operations – Sequencing Operations
- Members of the Wellcome Sanger Institute Tree of Life Core Informatics team
- Members of the Tree of Life Core Informatics collective
- Members of the Darwin Tree of Life Consortium
