# Supplementary material for: The genome sequence of the Small China-mark, Cataclysta lemnata (Linnaeus, 1758) (Lepidoptera: Crambidae)
Source: Wellcome Open Res. 2025 Nov 21;10:653. [Version 1] doi: 10.12688/wellcomeopenres.25182.1 (PMC12859424; doi:10.12688/wellcomeopenres.25182.1)
Supplement: Peer review history

# Reviewer report: Reviewer response for version 1

**DOI:** 10.21956/wellcomeopenres.27757.r144133

**Published:** 2026-02-04

**Authors:** Panagiotis Ioannidis

**Affiliations:** 1 Foundation for Research & Technology - Hellas, Crete, Greece

This paper describes the sequencing and assembly of the genome of the lepidopteran species, Cataclysta lemnata.

 The quality of the assembly is very high, as shown by the various metrics provided by the authors. Firstly, virtually the entire assembly is assigned to one of the 28 chromosome-level scaffolds. Secondly, k-mer completeness is >99%. Thirdly, QV = 59.9. Finally, complete BUSCOs = 96.6%, even though other genomes have a slightly better score (>99%), this is an acceptable BUSCO score as well.

 The blob plot (Figure 6) shows that there are some scaffolds of lower coverage and lower size than the chromosome-level scaffolds. What are these scaffolds? Could the authors comment on this?

 I also saw that the authors provide (through Ensembl) a gene set for this genome. However, no evaluation of this gene set is provided. I would at least expect a BUSCO evaluation in order to compare it with the BUSCO evaluation of the assembly; this way the interested researcher can tell how good the gene set actually is. Perhaps, for example, it is missing conserved genes. Or maybe all conserved genes are predicted correctly, but it's missing other, less conserved genes which are, nevertheless, essential for the organism.

 However, without a proper evaluation, one cannot know if he/she should use the provided gene set or if it's more worth investing time to generate a new (and more correct) gene set. Ensembl should definitely provide such an evaluation, but if it doesn't then it's up to the authors to do it. Running BUSCO is not such a difficult thing to do...

Are sufficient details of methods and materials provided to allow replication by others?

Yes

Is the rationale for creating the dataset(s) clearly described?

Yes

Are the datasets clearly presented in a useable and accessible format?

Yes

Are the protocols appropriate and is the work technically sound?

Yes

Reviewer Expertise:

bioinformatics, insect genomics

I confirm that I have read this submission and believe that I have an appropriate level of expertise to confirm that it is of an acceptable scientific standard.

# Reviewer report: Reviewer response for version 1

**DOI:** 10.21956/wellcomeopenres.27757.r145054

**Published:** 2026-02-03

**Authors:** Yvonne Jing Mei Liew

**Affiliations:** 1 Universiti Malaya, Kuala Lumpur, Kuala Lumpur, Malaysia

Background

- Please correct the following sentences.

-In Scotland, it occurs mainly in the southwest

 -TWe present a chromosomally complete genome sequence for C. lemnata.

 -example of Lepidoptera that can be used in studies of plastic waste degradation

- Please verify the subfamily of C. lemnata. In the Background section Acentropinae was used but in the Species taxonomy Nymphulinae was used.
- In this statement, an old Dutch reference from 1991 was cited but there is actually newer reference available from year 2025. Please consider the provided reference.

Their larvae can eat holes in PVC foil, providing another example of Lepidoptera that can be used in studies of plastic waster degradation (van der Velde, 1991).

 Gallitelli, L., Ceschin, S., Mariani, F., Pietrelli, L., & Scalici, M. (2025). Preliminary Observations on the Use of Microplastics by Aquatic Larvae of the Moth Cataclysta lemnata (Linnaeus, 1758). Environments, 12(3), 80. https://doi.org/10.3390/environments12030080

- chromosomally complete genome sequence

Please replace “chromosomally complete genome sequence” with appropriate terms such as chromosomal-level scaffolds. This is because although 99.51% of the assembly is assigned to 28 chromosomal scaffolds, the final assembly still contains 137 scaffolds and 874 gaps. The description chromosomally complete would imply gap-free chromosomes.

 Methods - Sample acquisition and DNA barcoding

 Please elaborate on where the tissue sample was dissected in the following section.

 -A small sample was dissected from the specimen and stored in ethanol

 Genome sequence report – Table 4

 The reported contig N50 length of 0.99 Mb does not meet the stated benchmark (≥1 Mb). Please provided an explanation if this is acceptable in the main text.

Are sufficient details of methods and materials provided to allow replication by others?

Yes

Is the rationale for creating the dataset(s) clearly described?

Yes

Are the datasets clearly presented in a useable and accessible format?

Yes

Are the protocols appropriate and is the work technically sound?

Yes

Reviewer Expertise:

Biochemistry

I confirm that I have read this submission and believe that I have an appropriate level of expertise to confirm that it is of an acceptable scientific standard.

# Reviewer report: Reviewer response for version 1

**DOI:** 10.21956/wellcomeopenres.27757.r145052

**Published:** 2026-02-03

**Authors:** Melody Clark

**Affiliations:** 1 Natural Environment Research Council, Cambridge, UK

This article describes the genome sequencing of a male specimen of the small China-mark moth. The biology and distribution of this species is well described, justifying it's inclusion in the Darwin Tree of Life sequencing project. The potential uses of the genome sequence, beyond taxonomy, including the fact that the larvae can eat holes in PVC and that the genome could be used to understand the properties of underwater silk were also explored. The procedures for isolating very small amounts of DNA, the production of libraries, sequencing strategies and assemblies are well described and follow standard Darwin Tree of Life protocols, which are all open source and freely available. The genome is of a very high standard resulting in 28 scaffolds with 27 autosomes and the Z sex chromosome. All sequencing data are freely available.

Are sufficient details of methods and materials provided to allow replication by others?

Yes

Is the rationale for creating the dataset(s) clearly described?

Yes

Are the datasets clearly presented in a useable and accessible format?

Yes

Are the protocols appropriate and is the work technically sound?

Yes

Reviewer Expertise:

Genomics and transcriptomics of invertebrates and fish.

I confirm that I have read this submission and believe that I have an appropriate level of expertise to confirm that it is of an acceptable scientific standard.

# Reviewer report: Reviewer response for version 1

**DOI:** 10.21956/wellcomeopenres.27757.r145051

**Published:** 2026-02-02

**Authors:** Xiaoguang Liu

**Affiliations:** 1 Henan Agricultural University, Zhengzhou, China

As a key component of the Darwin Tree of Life Project, this study achieves the first chromosome-level genome assembly of *Cataclysta lemnata*, effectively addressing the genomic resource gap for this species. The research background is articulated with clarity, detailing the species' taxonomic status, morphological characteristics, ecological habits, and distribution range, while underscoring the scientific value of genomic data in elucidating its aquatic adaptation, phylogenetic relationships, and unique biological traits. The experimental design is rigorous, employing PacBio HiFi long-read sequencing in conjunction with Hi-C chromatin conformation capture technology, ensuring sufficient sequencing depth, standardized assembly procedures, and comprehensive data analysis. The overall technical approach adheres to the high standards of contemporary genomics research. The genome assembly exhibits excellent quality, with a chromosome anchoring rate of 99.51% and BUSCO completeness reaching 96.6%. All core metrics conform to the standards for eukaryotic reference genomes, providing robust data support for subsequent related research. The paper presents a well-structured framework and clear data presentation, complying with open science research indexing standards, and demonstrates significant scientific value and application prospects.

   It is recommended that the authors supplement the details of their genome annotation. The manuscript currently only mentions the protein-coding gene annotation conducted using BRAKER2, which predicts a total of 17,004 protein-coding genes. However, it lacks comprehensive annotation information, including functional annotation results (such as Gene Ontology (GO), Kyoto Encyclopedia of Genes and Genomes (KEGG), and Pfam), as well as non-coding RNA annotations (e.g., microRNA (miRNA) and transfer RNA (tRNA)). To enhance the utility of the genomic resources presented, it is advisable to provide a complete genome annotation report that details the annotation pipeline and the core databases utilized.

Are sufficient details of methods and materials provided to allow replication by others?

Yes

Is the rationale for creating the dataset(s) clearly described?

Yes

Are the datasets clearly presented in a useable and accessible format?

Yes

Are the protocols appropriate and is the work technically sound?

Yes

Reviewer Expertise:

Physiological Toxicology, Insect Genomics and Evolutionary Research.

I confirm that I have read this submission and believe that I have an appropriate level of expertise to confirm that it is of an acceptable scientific standard.

# Reviewer report: Reviewer response for version 1

**DOI:** 10.21956/wellcomeopenres.27757.r144127

**Published:** 2026-01-30

**Authors:** Stephan Koblmüller

**Affiliations:** 1 University of Graz, Graz, Graz, Austria

This data note by Lees et al. presents the genome sequence of the small China-mark, *Cataclysta lemnata*. The authors provide comprehensive species background information and detailed methodologies covering specimen collection, sequencing, assembly, and quality assessments. I have no major concerns but have only two minor comments:

1. Background - Barcode / BIN: What exactly do you mean to say here; that the same haplotype as the one you sequenced is found across Europe, or that this applies to several haplotypes (not just the one you sequenced)? So, maybe clarify this by slightly rephrasing this sentence. And, in general, isn’t this a result of the study that actually belongs into a Results section?
2. Background: Change “TWe present a chromosomally …” to “We present a chromosomally …”

Are sufficient details of methods and materials provided to allow replication by others?

Yes

Is the rationale for creating the dataset(s) clearly described?

Yes

Are the datasets clearly presented in a useable and accessible format?

Yes

Are the protocols appropriate and is the work technically sound?

Yes

Reviewer Expertise:

phylogenetics/-genomics, population genenetics

I confirm that I have read this submission and believe that I have an appropriate level of expertise to confirm that it is of an acceptable scientific standard.

# Reviewer report: Reviewer response for version 1

**DOI:** 10.21956/wellcomeopenres.27757.r145049

**Published:** 2026-01-27

**Authors:** Christophe Klopp

**Affiliations:** 1 INRAE, Castanet-Tolosan, France

The authors present the first genome assembly of  the Small China-mark, *Cataclysta lemnata. *The sequencing protocol and bioinformatic pipelines used are standard. 

 The authors should include the Hi-C data in the hifiasm assembly as this is best practice to produce phased contigs and chromosomes. 

 The authors should provide hap2 in chromosomes this will ease the work of biologists interested in the species, for example to find variations between haplotypes. 

 Figure 4. (Evaluation of *k*-mer completeness using MerquryFK.) show that some haplotype share kmers are missing in the primary assembly. This should be discussed. 

 Why are the genomescope2 and merqury kmer plot not representing the same proportions between specific and shared kmers? There seem to be much less specific kmers found by merqury.

 "TWe present a" should be replaced by "We present a"

Are sufficient details of methods and materials provided to allow replication by others?

Yes

Is the rationale for creating the dataset(s) clearly described?

Yes

Are the datasets clearly presented in a useable and accessible format?

Yes

Are the protocols appropriate and is the work technically sound?

Yes

Reviewer Expertise:

genome assembly and annotation

I confirm that I have read this submission and believe that I have an appropriate level of expertise to confirm that it is of an acceptable scientific standard, however I have significant reservations, as outlined above.

# Reviewer report: Reviewer response for version 1

**DOI:** 10.21956/wellcomeopenres.27757.r145050

**Published:** 2026-01-22

**Authors:** Laurence Despres

**Affiliations:** 1 Laboratoire d'Ecologie Alpine (Ringgold ID: 56837), Grenoble, Auvergne-Rhône-Alpes, France

The authors report on the chromosome-level genome assembly of a male specimen of the Small China-mark, *Cataclysta lemnata* (Lepidoptera; Crambidae) a common moth found in freshwater habitats including ponds and lakes throughout the Palaearctic. The genome sequence has a total length of 432.44 megabases. The assembly contains two haplotypes with most of haplotype 1 scaffolded into 28 chromosomes, including the Z sex chromosome. The mitochondrial genome has also been assembled.

 The quality of the assembly was further assessed by the proportion of complete BUSCO genes recovered, which is very high : 96.6% of the expected gene set of lepidoptera_odb10 reference set ( *n* = 5 286). The genome assembly is of good quality and was constructed using appropriate methods: Pac Bio HiFi long reads (71 X coverage) and Hi-C data to confirm scaffolding, following the high standard of the Darwin Tree of Life Project for results presentation. The BlobToolKit snail plot provides an overview of assembly metrics and BUSCO gene completeness.

Are sufficient details of methods and materials provided to allow replication by others?

Yes

Is the rationale for creating the dataset(s) clearly described?

Yes

Are the datasets clearly presented in a useable and accessible format?

Yes

Are the protocols appropriate and is the work technically sound?

Yes

Reviewer Expertise:

evolutionary genomics

I confirm that I have read this submission and believe that I have an appropriate level of expertise to confirm that it is of an acceptable scientific standard.
